# Inhibitory effects of saeu‐jeot extract on NLRP3 inflammasome activation and radiation‐induced micronucleus formation

**DOI:** 10.1002/fsn3.2808

**Published:** 2022-04-27

**Authors:** Lan Li, Hee‐Sun Kim, Sung‐Won Kwon, Min‐Jae Lee

**Affiliations:** ^1^ 26453 School of Public Health and Management Wenzhou Medical University Wenzhou China; ^2^ 34962 Department of Laboratory Animal Medicine College of Veterinary Medicine Kangwon National University Chuncheon South Korea; ^3^ Radiation Health Research Institute Korea Hydro and Nuclear Power Co., Ltd. Seoul South Korea; ^4^ CHA Bundang Medical Center CHA University Seongnam‐si South Korea

**Keywords:** inflammasome, micronuclei, NLRP3, radiation, saeu‐jeot

## Abstract

**Purpose:**

Saeu‐jeot, or shrimp paste, is a traditional Korean high‐salt fermented seafood. This study elucidated the effects of saeu‐jeot extract (SJE) on NLRP3 inflammasome activation and radiation‐induced micronucleus formation.

**Method:**

We treated lipopolysaccharide‐primed mouse bone marrow‐derived macrophages with the NLRP3 inflammasome activators ATP and nigericin. We also analyzed the acute effects of ionizing radiation on micronucleated polychromatic erythrocytes (MnPCEs) in whole‐body gamma‐irradiated male BALB/c and C57BL/6 mice after oral administration of SJE.

**Results:**

SJE significantly inhibited NLRP3 inflammasome activation, reducing IL‐1β secretion in vitro. In addition, the frequency of MnPCEs was significantly lower in SJE‐treated mice.

**Conclusions:**

SJE has anti‐inflammatory effects and reduces radiation‐induced chromosome damage.

*Advance in knowledge*: There are no reports concerning the effects of SJE on NLRP3 inflammasome activation or radioprotection. This experiment showed the radioprotective effects of saeu‐jeot.

## Graphical abstract

SJE exerts an anti‐inflammatory effect and reduces radiation‐induced chromosomal damage.

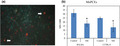

## 1 INTRODUCTION

Human exposure to ionizing radiation is increasing with clinical and technological developments. The development of radioprotective agents is important to protect patients from the side effects of radiotherapy and the public from unwanted irradiation (Yamini & Gopal, 2010). The harmful effects of ionizing radiation are presumably mediated by the interaction of free radicals with DNA or other cellular macromolecules (Wiseman & Halliwell,). Radiation‐induced ROS and the resulting inflammation are increasingly recognized as key players in radiation damage, particularly at the chromosome level (Multhoff & Radons, 2012). The NOD‐like receptor (NLR) family pyrin domain‐containing 3 (NLRP3) inflammasome is the most fully characterized inflammasome; it consists of the NLRP3 scaffold, apoptosis‐associated speck‐like protein containing a CARD (ASC) adaptor, and caspase‐1 (Jin & Flavell, 2010). The NLRP3 inflammasome is a cytoplasmic protein complex that mediates inflammatory responses to a broad array of signals. Pathogen‐ and damage‐associated molecular pattern (PAMP and DAMP) molecules and environmental irritants can activate NLRP3 (Jin & Flavell, 2010). By means of the adaptor protein ASC, NLRP3 recruits and activates caspase‐1, leading to the cleavage of precursors of the pro‐inflammatory cytokines IL‐1β and IL‐18 (Ozaki et al., 2015). IL‐1β is an extremely important cytokine because of its central role in the inflammatory process.

Gamma radiation can increase ROS levels, resulting in IL‐1β expression. ROS are presumed to activate NLRP3 by promoting the dissociation of thioredoxin‐interacting protein (TXNIP) from thioredoxin (TRX), which allows TXNIP to bind directly to and activate NLRP3 (Haneklaus et al., 2013; Licandro et al., 2013). Inflammation has a pivotal role in modulating radiation responsiveness; inflammatory mediators act together to perpetuate and amplify the inflammatory cascade. These mediators suppress DNA repair, leading to microsatellite and chromosome instability, which culminate in abnormal chromosome segregation and aneuploidy (Multhoff & Radons, 2012). Micronucleus formation is a sign of this abnormal chromosome segregation. We previously studied the inhibition of radiation‐induced micronucleus formation by Korean traditional fermented soybean food (Kim et al., 2019). Saeu‐jeot, a traditional Korean high‐salt fermented seafood, consists of tiny shrimp that have been fermented for several months with sea salt. Recently, the therapeutic effects of SJE have been evaluated (Koo et al., 2016); however, there are no reports concerning the effects of SJE on NLRP3 inflammasome activation or radioprotection. Therefore, this study assessed the effects of SJE on NLRP3 inflammasome activation in vitro and on radiation‐induced micronucleus formation in vivo.

## 2 MATERIALS AND METHODS

### 2.1 Differentiation of bone marrow‐derived macrophages (BMDMs)

Primary BMDMs were cultured and differentiated in vitro; treated lipopolysaccharide‐primed mouse BMDMs with the NLRP3 inflammasome activators ATP and nigericin (NG). Briefly, BMDMs were derived from femoral and tibial bone marrow progenitors. The progenitors were cultured in Dulbecco's modified Eagle medium (DMEM; PAA Laboratories, GE Healthcare Bio‐Sciences Co., NJ, USA) supplemented with 10% fetal bovine serum (FBS; PAA, GE Healthcare Bio‐Sciences Co.), 100 µg/ml penicillin/streptomycin (PAA, GE Healthcare Bio‐Sciences Co.), and 30% L929 cell‐conditioned medium (LCCM) as a source of granulocyte/macrophage colony‐stimulating factor. Cells were cultured at 37°C in a 5% CO2 incubator for 7 days.

### 2.2 NLRP3 inflammasome activation

BMDMs (1.0 × 10^6^ cells per well) were plated in 12‐well plates and primed with 10 µg/ml lipopolysaccharide (LPS; Sigma‐Aldrich Co., MO, USA) in RPMI 1640 medium for 3 h. After LPS priming, NLRP3 was activated in the BMDMs using one of several reagents. ATP (2 mM; InvivoGen, CA, USA), NG (40 µM; Tocris Bioscience, Bristol, UK), aluminum potassium sulfate (Alum, 2 mg/ml; Daejung Chemicals & Metals, Gyenggi‐do, Korea), or CaCl_2_ (1 mM; Biosesang, Seoul, Korea) were used as NLRP3 inflammasome activators by introducing them 1 or 3 h before the supernatant was collected. To examine the inhibitory effect of SJE on inflammasome activation, the extract (provided by the Korea Food Research Institutes, Gyeonggi‐do, Korea) was introduced with the above activators. Supernatants and lysates were collected for further analysis.

### 2.3 Western blot analysis

Supernatants, lysates, and pellet samples were separated by SDS‐PAGE using 10% or 16% polyacrylamide gels. After electrophoresis, the separated proteins were transferred to PVDF membranes (Pall Co., NY, USA). The membranes were probed overnight at 4°C with anti‐mouse IL‐1β antibody (R&D Systems, MN, USA) or anti‐actin antibody (Santa Cruz Biotechnology, CA, USA). The membranes were further probed with HRP‐conjugated secondary anti‐sera (Santa Cruz Biotechnology) and visualized by ECL solution (Millipore, MA, USA) and a cooled CCD camera system (ATTO Technology, Tokyo, Japan).

### 2.4 Animals

All experiments were carried out with 6‐ to 8‐week‐old male BALB/c and C57BL/6 mice weighing 19–23 g (Orient Bio, Seongnam‐Si, Gyeonggi‐Do, Korea). These animals were bred and maintained at 25 ± 2°C on the standard mouse diet (LabDiet, St. Louis, MO, USA). All animal experiments were approved by the Kangwon National University Institutional Animal Care and Use Committee (KIACUC‐130325‐1).

### 2.5 Preparation of SJE

SJE was prepared by Korea Food Research Institute. Saeu‐jeot (1 kg) was mixed with ethanol (99.99%) and suspended overnight at 25°C. It was filtered through a coarse filter (No. 1, Advantec) and concentrated by vacuum evaporation. The concentrate was filtered a second time through a fine filter (No. 2, Advantec). The filtrate was adjusted to pH 7.4 with 1.0 or 0.1 mol/L NaOH. The concentration of the final SJE was adjusted to 100 mg/ml with distilled water.

### 2.6 Treatment of mice with SJE

A total of 200 mg of prepared SJE/kg body weight was administered by gavage (2 ml/kg per each diluted with 8 ml/kg normal saline) to the experimental animals for 6 weeks (once a day, 5 days a week). The animals were irradiated a day after the final feeding.

### 2.7 Irradiation

Whole‐body irradiation of the experimental animals was carried out in a gamma chamber (Gammacell 40 Exactor, Ottawa, Ontario, Canada) obtained from Radiation Health Research Institute, Korea Hydro and Nuclear Power Co., Ltd. Animals were exposed to 2‐Gy gamma radiation.

### 2.8 Body and spleen weight

In order to monitor any changes at the whole‐body level, we weighed mice immediately following irradiation. In this study, body weight is expressed as the rate of weight gain after irradiation. Spleen weight is expressed as a proportion of body weight (spleen mass [g]/body mass [g]).

### 2.9 Measurement of white and red blood cells and platelets

Following irradiation, blood was collected from the subclavian vein under ether anesthesia. Whole blood was collected using an EDTA‐containing tube (SARSTEDT, Germany); and the number of white blood cells, red blood cells, and platelets were counted using an automatic cell counter (SEAC, Italy).

### 2.10 Micronuclei analysis

Micronucleus assay was used to assess chromosome damage as described previously (Kim et al., 2019). Briefly, acridine orange (Merck, Germany) was dissolved in distilled water (1 mg/ml), and 10 µl of this solution was placed on pre‐heated (~70°C) clean glass microscope slides. Approximately 3 µl of peripheral blood was obtained from the subclavian vein and smeared onto a glass slide. MnPCEs was analyzed by a fluorescence microscope, and at least 1,000 polychromatic erythrocytes in the peripheral blood were scored per mouse for each data point. Five mice were included in each experimental group.

### 2.11 Statistical analysis

All data are presented as the mean ± standard deviation. For comparisons of MnPCEs between groups, data were analyzed using the SAS 9.3 statistical package. All data were analyzed using the nonparametric Kruskal–Wallis test.

## 3 RESULTS

### 3.1 Effects of SJE on body and spleen weights and blood cells

There was no significant difference in the rate of body weight gain and spleen‐to‐body weight between the mice after irradiation with and without SJE extract (Table 1).

**TABLE 1:** Rate of gain and relative spleen weights in mice after irradiation with and without SJE

Treatment	BALB/c		C57BL/6	
	Control	SJE	Control	SJE
Rate of gain	1.01 ± 0.01	0.97 ± 0.02	0.98 ± 0.03	0.97 ± 0.01
Spleen (g% B.W.)	0.25 ± 0.03	0.25 ± 0.02	0.20 ± 0.02	0.22 ± 0.02

Note

Data are presented as mean ± SD.

Hematological test results showed that there was no significant difference in leukocytes, erythrocytes, and platelets between control group and SJE group (Table 2).

**TABLE 2:** Hematological analyses of mice after irradiation with and without SJE

Treatment	BALB/c		C57BL/6	
	Control	SJE	Control	SJE
WBC(10^^^9/L)	0.65 ± 0.39	0.60 ± 0.41	0.48 ± 0.25	0.43 ± 0.30
RBC(10^^^12/L)	9.08 ± 0.33	9.72 ± 0.93	9.17 ± 1.18	10.14 ± 1.03
PLT(10^^^9/L)	354.5 ± 87.92	396 ± 99.70	458 ± 101.77	585.6 ± 197.02

Note

Data are presented as mean ± SD.

Abbreviations: PLT, platelet; RBC, red blood cell; WBC, total white blood cell.

### 3.2 SJE inhibits NLRP3 inflammasome activation

**FIGURE 1:**
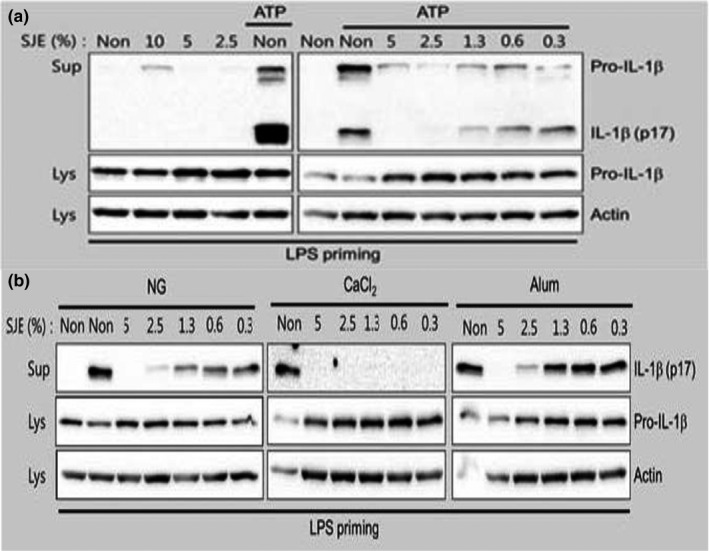
Effects of SJE on NLRP3 inflammasome activation. BMDMs were primed by LPS in RPMI medium containing 10% FBS and antibiotics for 3 h. Cells were supplied with RPMI medium in the present with the indicated percentages of SJE with/without NLRP3 inflammasome activators, ATP, nigericin (NG), and CaCl_2_ for 1 h, or aluminum potassium sulfate (Alum) for 3 h, and were analyzed for IL‐1β secretion. Cell culture supernatants (Sup) and cell lysates (Lys) were analyzed by immunoblot assay as indicated

To assess the effects of SJE alone on NLRP3 inflammasome activation, various dosages of extract (2.5%–10%) were used to treat LPS‐primed BMDMs. No mature IL‐1β was detected in the supernatant, although secreted pro‐IL‐1β was observed with the higher concentrations of SJE. Instead, we assessed the inhibitory effects of SJE on inflammasome activation by various known activators. Surprisingly, SJE inhibited IL‐1β secretion after activating BMDMs with a well‐characterized NLRP3 inflammasome activator ATP (Figure 1a). In addition, we elucidated the inhibitory effects of SJE on NLRP3 inflammasome activation with other NLRP3 activators, such as nigericin (NG), calcium chloride (CaCl_2_), and aluminum potassium sulfate (Alum). SJE attenuated the production of mature IL‐1β resulting from NG‐, CaCl_2_‐, and Alum‐induced NLRP3 inflammasome activation, although the degree of inhibition by SJE varied (Figure 1b).

### 3.3 Sodium and magnesium within SJE inhibit NLRP3 inflammasome activation

**FIGURE 2:**
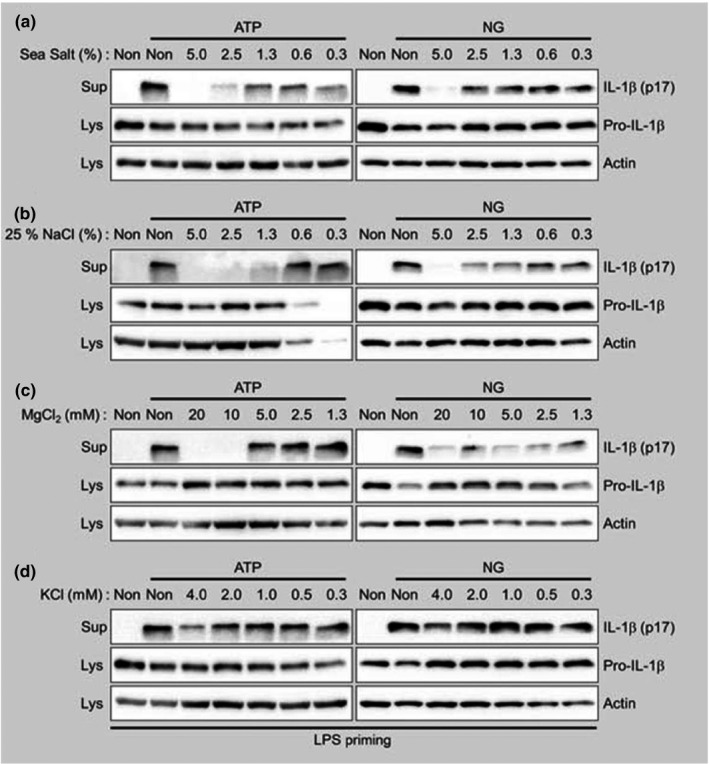
Effects on the sea salt and its ingredients of SJE on the NLRP3 inflammasome activation. LPS‐primed BMDMs were stimulated with NLRP3 inflammasome activator, ATP or nigericin (NG), in the presence of the indicated concentration of sea salt (a), NaCl (b), MgCl_2_ (c), or KCl (d). After 1 h, cell culture supernatants (Sup) and cell lysates (Lys) were collected and analyzed by immunoblot assay as indicated

To identify the SJE component responsible for inhibiting NLRP3 inflammasome activation, we focused on the sea salt because it is the major (around 25% w/w) component of the saeu‐jeot. The sea salt (manufactured by solar evaporation) is mainly composed of NaCl (81.75%) but contains additional minerals, such as magnesium (9,645 mg/kg) and potassium (3,456 mg/kg), based on the 2010 International Solar Salt Fair (Gwangju, Korea). Thus, we treated LPS‐primed BMDMs with sea salt, at 25% of the saeu‐jeot dosage, in the presence of NLRP3 inflammasome activators (ATP and NG). As shown in Figure 2, like SJE, sea salt inhibited IL‐1β secretion from ATP‐ or NG‐treated BMDMs. In addition, NaCl also blocked IL‐1β maturation resulting from NLRP3 inflammasome activation. One of the major mineral components, magnesium, also inhibited IL‐1β production. However, KCl did not alter IL‐1β maturation induced by ATP or NG (Figure 2d). Thus, the sodium and magnesium in the sea salt, the major component of saeu‐jeot, inhibit NLRP3 inflammasome activation.

### 3.4 Frequency of micronuclei

**FIGURE 3:**
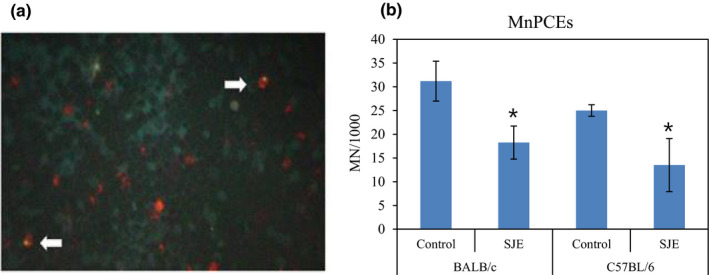
Polychromatic erythrocyte staining and micronucleus analysis. (a) Acridine orange‐stained peripheral blood erythrocytes of mouse. Arrow indicates micronucleus in polychromatic erythrocyte. (b) In both mouse strains experiments, the frequency of radiation‐induced micronuclei polychromatic erythrocytes (MnPCEs) was significantly lower in SJE group than that control (SJE group: mean = 18.25 ± 3.49, control group: mean = 31.2 ± 4.21, BALB/c mice; SJE group: mean = 13.5 ± 5.59, control group: mean = 25 ± 1.22, C57BL/6 mice. *p < .05, *n* = 5 mice/group). SJE, Saeu‐Jeot's extracts

Micronuclei are additional small nuclei formed by excluding chromosome fragments or the whole chromosome during mitosis. Therefore, the micronucleus rate reflects chromosome breakage mitotic apparatus damage. Based on this, we compared the MnPCEs frequencies between treatment and control groups. There was a significant difference between control and SJE treatment in both mouse strains (*p < .05) (Figure 3).

## 4 DISCUSSION

### 4.1 Inflammatory responses induced by irradiation cause chromosome damage

Radiation‐induced inflammatory responses have been proposed to affect chromosome instability (Licandro et al., 2013; Multhoff & Radons, 2012). The inflammasome drives the cleavage of the inflammatory cytokine precursor pro‐IL‐1β into the active form IL‐1β, which can then be secreted and induce inflammatory responses that lead to DNA damage (Multhoff & Radons, 2012; Wiseman & Halliwell,). During this process, inflammasomes NLRP3, NLRC4, and AIM2 have key roles in the activation of inflammatory cytokine precursors (Jin & Flavell, 2010). The NLRP3 inflammasome drives DNA damage after oxidative and genotoxic stress; it sustains the ROS‐mediated oxidation of DNA (Licandro et al., 2013).

### 4.2 SJE inhibited NLRP3 inflammasome activation

In this study, we observed that SJE inhibited IL‐1β secretion after the activation of BMDMs with a well‐characterized NLRP3 inflammasome activator. We also elucidated the inhibitory effects of SJE on NLRP3 inflammasome activation by other NLRP3 activators, such as NG, CaCl_2_, and Alum. SJE attenuated mature IL‐1β production resulting from NG‐, CaCl_2_‐, and Alum‐induced NLRP3 inflammasome activation, thereby inhibiting NLRP3 inflammasome activation, although the degree of inhibition varied. Similar to SJE, sea salt inhibited IL‐1β secretion from ATP‐ or NG‐treated BMDMs. NaCl also blocked the IL‐1β maturation resulting from NLRP3 inflammasome activation. Magnesium, a major mineral in sea salt, inhibited IL‐1β production. However, potassium (KCl), which is an NLRP3 inflammasome inhibitor, did not alter the IL‐1β maturation induced by ATP or NG. Therefore, the sodium and magnesium in sea salt, a major component of saeu‐jeot, inhibit NLRP3 inflammasome activation.

### 4.3 SJE treatment reduced MnPCE frequency

There is within‐species genetic variation in radiation sensitivity. The range of radiation LD_50/30_ values ranges from 4 to 6.5 Gy among laboratories; it is confounded by differences in the age at exposure, radiation quality, and unidentifiable environmental factors (Grahn & Hamilton, 1957; Hamasaki et al., 2007). BALB/c mice are considered more radiosensitive than C57BL/6 mice; the difference in LD_50/30_ values between the two strains is approximately 1.3 Gy (Hamasaki et al., 2007).

As expected, there were significantly more radiation‐induced MnPCEs in BALB/c mice than in C57BL/6 mice in both the SJE‐treated and untreated groups. The SJE‐treated groups did not show significant differences in body weight gain, relative spleen weight, hematology, or any gross histopathological changes, compared with the untreated groups. Despite the genetic differences in radiosensitivity, there were significantly fewer MnPCEs in the SJE treatment groups than in the controls for both strains, indicating that SJE has radioprotective effects in both radiosensitive (BALB/c) and radioresistant (C57BL/6) mice. With respect to the MnPCE result, 2 Gy was an adequate radiation dose in this study because both strains showed irradiation effects and clear genetic differences.

### 4.4 Anti‐oxidative effects of the ingredients in SJE

Other factors may have contributed to the decrease in MnPCEs in the SJE treatment groups because MnPCEs mainly result from cellular oxidative stress (Iyer & Lehnert, 2000). Chitosan, a natural nontoxic biopolymer produced by the deacetylation of chitin, is a major component of crustacean shells (e.g., crabs, shrimp, and crawfish). Chito‐oligosaccharides (COS) are the enzymatic or chemical hydrolysis products of chitosan or chitin; they may be generated during chitosan fermentation. Many recent studies have reported that chitin and its derivatives, such as COS, inhibit DNA and protein oxidation. In addition, intracellular glutathione levels and direct intracellular radical scavenging by mouse macrophages were increased in the presence of chitin and chitin derivatives, exerting an overall protective effect against cellular oxidative stress (Lodhi et al., 2014; Ngo et al., 2009; Park & Kim, 2010). During the 6‐week SJE treatment, oxidative stress in hematopoietic tissue was decreased, which could reduce radiation‐induced chromosome damage. Some ingredients in SJE are free radical scavengers that reduce the damage caused by ionizing radiation‐induced free radicals. Further studies are necessary to determine which ingredients contribute to the radioprotective effects of saeu‐jeot and to elucidate the mechanisms by which such effects are mediated.

### 4.5 SJE is a radioprotective agent

To be clinically useful, a good radioprotective agent must be effective, readily available, specific, and well tolerated (Duraković, 1993). Saeu‐jeot, a fermented seafood commonly consumed in Korea, is an inexpensive, readily available agent that can be used for an extended period of time; thus, it may serve as a good radioprotective agent. Our results support this application.

## CONFLICT OF INTEREST

The authors declare that there are no potential conflicts of interest regarding the publication of this article.

## Data Availability

All datasets obtained and analyzed during this study are available from the corresponding author on reasonable request.
